# Eosinophilic gastrointestinal disorders in patients with inborn errors of immunity: Data from the USIDNET registry

**DOI:** 10.3389/fimmu.2022.987895

**Published:** 2022-09-23

**Authors:** Paulina Tran, Laura Gober, Elizabeth K. Garabedian, Ramsay L. Fuleihan, Jennifer M. Puck, Kathleen E. Sullivan, Jonathan M. Spergel, Melanie A. Ruffner

**Affiliations:** ^1^ Division of Allergy & Immunology, Children’s Hospital of Philadelphia, Philadelphia, PA, United States; ^2^ National Human Genome Research Institute, National Institutes of Health, Bethesda, MD, United States; ^3^ Division of Allergy & Immunology, Columbia University Irving Medical Center, New York, NY, United States; ^4^ Division of Allergy, Immunology and Blood and Marrow Transplantation, Department of Pediatrics, UCSF Benioff Children’s Hospital San Francisco, School of Medicine, University of California San Francisco, San Francisco, CA, United States; ^5^ Department of Pediatrics, Perelman School of Medicine at the University of Pennsylvania, Philadelphia, PA, United States

**Keywords:** primary immunodeficiency, eosinophilic gastrointestinal disorders (EGID), eosinophilic esophagitis (EoE), inborn errors of immunity (IEI), immune dysregulation

## Abstract

**Rationale:**

Eosinophilic gastrointestinal disorders (EGID), including eosinophilic esophagitis (EoE), are inflammatory disorders of the gastrointestinal mucosa mediated by complex immune mechanisms. Although there have been initial reports of EGID in patients with inborn errors of immunity (IEI), little is known about the presentation of EGID in immunodeficient individuals.

**Methods:**

We queried the U.S. Immunodeficiency Network (USIDNET) for patient records including the terms eosinophilic esophagitis, gastritis, enteritis, or colitis. We analyzed 74 patient records from the database, including diagnoses, demographics, infectious history, laboratory findings, genetic studies, therapeutic interventions, and clinical outcomes.

**Results:**

We examined 74 patient records. A total of 61 patients had isolated EoE, and 13 had distal gastrointestinal involvement consistent with EGID. The most common IEI were common variable immunodeficiency (43.2%), some form of combined immunodeficiency (21.6%), chronic granulomatous disease (8.1%), hyper-IgE syndrome (6.8%), and autoimmune lymphoproliferative syndrome (6.8%). The median age at presentation with IEI was 0.5 years (IQR 1.725, max 39 years) and 56.76% were male. Approximately 20% of the patients in the cohort received a hematopoietic stem cell transplantation for treatment of IEI, but the timing of the HSCT in relationship to the EGID diagnosis was unknown.

**Conclusions:**

Here, we report EGID in a diverse cohort of IEI patients, suggesting that both non-EoE EGID and EoE can be seen as comorbid conditions with a variety of IEI. Our data suggests that EGID may be more common in patients with IEI than would be expected based on estimates of EGID in the general population.

## Introduction

Inborn errors of Immunity (IEI) are a heterogenous group of disorders that result in susceptibility to infection, autoinflammation, and immunodysregulation. The majority of IEI are genetic, with over 450 distinct genetic causes of IEI described to date (1). However, some forms of IEI, such as common variable immunodeficiency (CVID), are not attributable to a monogenic cause (2–4). IEI can affect diverse immunologic pathways, resulting in dysfunction of one or more immunologic mechanisms (1). Timely diagnosis of IEI decreases the time to definitive therapy and decreases morbidity and mortality. Therefore, it is critical to understand the various manifestations of IEI.

Patients with IEI may first present with signs of immune dysregulation, which can precede symptoms or lab findings indicative of immunodeficiency (5–7). These can include autoimmune or atopic manifestations. The specific risks for these manifestations vary based on the immunologic mechanisms that underlie the patient’s IEI. Allergic inflammation is dependent on effector cells and molecules and can be potentiated by a specific lack of regulatory mechanisms due to the underlying IEI. There has been an increasing understanding of which IEI carry an increased risk for specific atopic manifestations, with most studies focusing on atopic dermatitis, allergic rhinitis, asthma, and immunoglobulin E (IgE)-mediated food allergy. Although eosinophilic esophagitis (EoE) is thought to co-occur with these disorders following a common atopic pathophysiology (8), EoE and non-EoE eosinophilic GI disorders and their association with IEI are relatively poorly understood.

Eosinophil-associated gastrointestinal disorders (EGIDs) are characterized by a chronic inflammatory eosinophilic infiltration of the gastrointestinal tract and include EoE, eosinophilic gastritis, eosinophilic gastroenteritis, eosinophilic enteritis, and eosinophilic colitis. EoE is estimated to affect approximately 1 in 1-2000 persons (9) and is characterized by an eosinophilic infiltrate greater than 15 eosinophils per high-powered field (~60 eos/mm^2^) into the mucosa with chronic symptoms of esophageal dysfunction, such as vomiting, food refusal, dysphagia, odynophagia, food impaction, or strictures. The prevalence of non-EoE EGID in the general population is estimated at 3–8/100,000 cases and is associated with symptoms of abdominal pain, nausea, vomiting, decreased appetite, diarrhea, and weight loss (10). The diagnostic eosinophil count on biopsy varies depending on the location in the gastrointestinal tract (10). The association of EGID and EoE in IEI patients has not been thoroughly investigated. However, there are reports of EoE in patients with IEI, including CVID, chronic granulomatous disease (CGD), and STAT1 gain of function (11–14). EGID, including esophageal, gastric, and colonic involvement, has been reported in patients with STAT 3 deficiency [autosomal dominant hyper immunoglobulin E syndrome (AD-HIES)] and PTEN hamartoma syndrome (15, 16).

The goal of this study was to understand which IEI patients may present with EGID. To investigate this, we queried IEI patient records from the United States immunodeficiency Network (USIDNET). USIDNET is a national registry that provides clinical and laboratory data on patients with IEI and provides crucial information for understanding IEI (17). Here, we identify EGIDs, including EoE, associated with a broader spectrum of IEI than had previously been appreciated.

## Materials and methods

### Ethics statement

USIDNET is an Immune Deficiency Foundation program and is an NIH-funded research consortium that maintains a patient-consented registry of data from IEI patients in North America. Patient data collection for USIDNET proceeds under the supervision of each enrolling institution’s Institutional Review Board. All patients provide informed consent for inclusion into the USIDNET database, which contains clinical, laboratory, and molecular data from IEI patients.

### US Immunodeficiency Network (USIDNET) data search

We performed a retrospective analysis of EGID in IEI reported in the USIDNET (date of data release April 15, 2020). The USIDNET search examined the records of all IEI patients containing a comorbid diagnosis of eosinophilic esophagitis, gastritis, enteritis, or colitis. Patients were included in the cohort if they were found to have one of these diagnoses. Gastrointestinal biopsy data and EGID disease status was not available in USIDNET.

Patients of all ages and underlying IEI diagnoses were included in this study. Two patient records were excluded from the analysis in the cohort due to diagnosis of unspecified eosinophilia without any mention EGID diagnosis, resulting in 74 total USIDNET records for the analysis.

The data from the USIDNET for analysis in the study included IEI diagnosis, patient age, sex, race, laboratory data, comorbid infections, and other medical conditions. Gastrointestinal biopsy eosinophil count data is not collected in the USIDNET and therefore not available for the patients in this cohort. Limited data regarding treatment with hematopoietic stem cell transplant as well as immunoglobulin replacement was available for a subset of patients.

Patient laboratory values, diagnoses, genetics, and treatment information are presented as reported in the database, including the overall category of IEI diagnosis.

The patient birth month and date were not provided to protect privacy, therefore we used June 15^th^ and the provided year of the patient birth to minimize error when imputing the patients’ age at the time of laboratory assessments. The statistical analyses were performed in Stata (StataCorp, Collegetown, TX) and Graphpad Prism (Graphpad Software Inc, La Jolla, CA).

### Assessment of atopy

We assessed patient records for diagnoses consistent with atopic disorders. Patients were considered to have asthma or eczema if these diagnoses were listed. Both listings of eczema and atopic dermatitis were considered positive for eczema. Patients were considered to have IgE-mediated food allergy if: (1) food allergy was listed as a condition or (2) a food allergen was listed to cause symptoms of IgE-mediated food allergy, such as hives/urticaria, stridor, wheezing, angioedema, or anaphylaxis. We did not include patients who avoided foods for gastrointestinal symptoms alone, EoE management, dermatitis, or unknown reasons.

### Assessment of infections

Patients were considered to have candidiasis if they were listed to have thrush, mucocutaneous candidiasis, or candidiasis of the esophagus.

## Results

Our query of the USIDNET registry resulted in a total of 74 patients with a diagnosis of EGID. Among the patients, 56.7% were male and 71.6% were White (**Table 1**). The age of IEI diagnosis was reported for 67.5% of the patients and ranged from newborn to 39 years old, with a median age of 7 years (interquartile range 2-16 years). Thirty patients (40.5%) of the cohort had no additional atopic diagnoses, but the remainder had at least one atopic comorbidity (**Table 1**). Eleven patients (14.9%) had some combination of two additional atopic conditions, and 7 patients (9.5%) had diagnoses of eczema, asthma, and food allergy.

**Table 1:** Demographic and clinical characteristics of EoE and EGID patients in USIDNET (N= 74).

Characteristic	Value
**Patient reported onset age,** median years (IQR) *data available for 50/74 patients*	0.5 (0.275-2)
**Age at diagnosis**, median years (IQR) *data available for 55/74 patients*	7 (2,16)
**Sex**, male, n (%)	42 (56.7)
**Race**, n (%)	
White/Caucasian	53 (71.6)
Black/African American	4 (5.4)
Asian or Pacific Islander	2 (2.7)
Unknown/Not reported	9 (12.2)
Other/More than one race	4 (5.4)
American Indian/Alaskan Native	2 (2.7)
**Ethnicity** (Hispanic/Latino), n (%)	
Yes	3 (4.0)
No	56 (75.7)
Unknown/Not reported	15 (20.3)
**Atopic Conditions**, n (%)	
Eczema	26 (35.1)
Asthma	29 (39.2)
IgE-mediated food allergy	14 (18.9)

**Figure 1:**
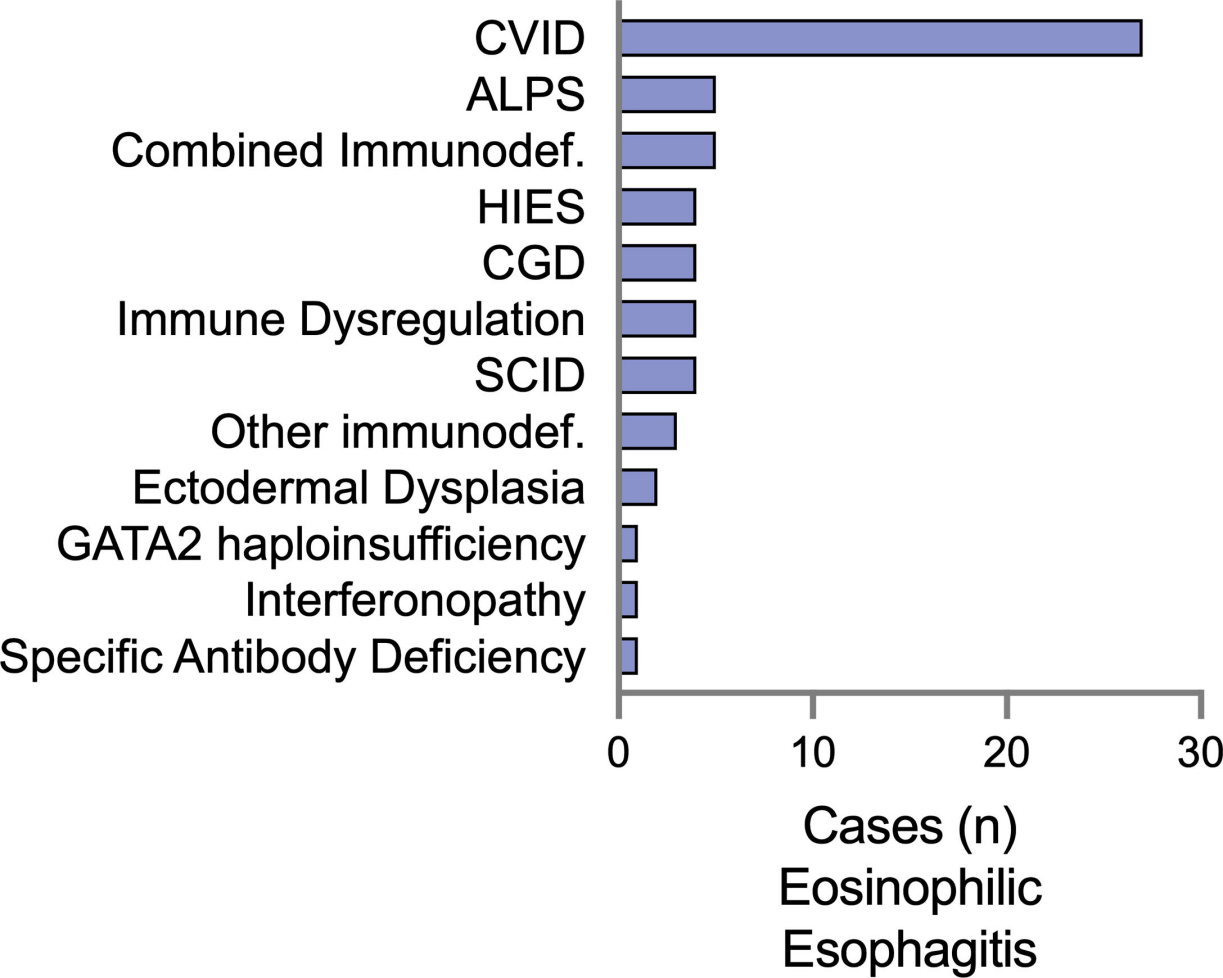
Eosinophilic esophagitis (EoE) is a comorbid diagnosis among a diverse set of IEI in the USIDNET. The number of patients with EoE as a listed condition are represented using the immunodeficiency category as defined by USIDNET at the time of data collection. CVID, Common variable immunodeficiency; CGD, chronic granulomatous disease; ALPS, autoimmune lymphoproliferative syndrome; HIES, Hyper-IgE syndrome; SCID, severe combined immunodeficiency; GATA2, GATA-binding factor 2.

We examined IEI diagnoses associated with EoE and EGID separately, with the rationale that there may be separate immunologic mechanisms that contribute to these distinct clinical entities. There were 61 patients diagnosed with EoE alone (**Figure 1**). The majority had underlying humoral defects, including 44.2% (27/61) with common variable immunodeficiency (CVID) and one patient with specific antibody deficiency. A specific genetic etiology of immunodeficiency was identified in 34.4% (21/61) of patients. These included patients presenting with severe combined immunodeficiency (SCID), representing 6.5% of the patients. Similar to previous reports, we observed an association with hyper-IgE syndrome (15), but with several reported genetic etiologies beyond STAT3, including PGM3 and DOCK8. Additional combined immunodeficiencies observed included activated PI3K-delta syndrome (PIK3CD 4/61) and 2 combined immunodeficiencies with no genetic mutation reported. Interestingly, 8% (4/61) were diagnosed with chronic granulomatous disease (CGD). Regarding atopic disease with the EoE subset, 32.8% of the patients were reported to have eczema, 39.3% had asthma, and 19.7% had IgE-mediated food allergies.

**Figure 2:**
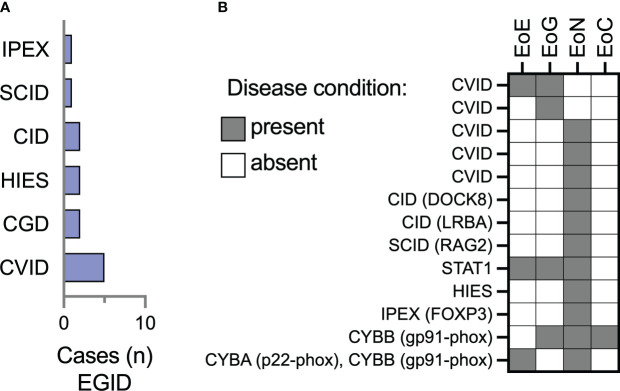
Eosinophilic enteritis is the most frequently represented site of extraesophageal GI eosinophilia among IEI patients in the USIDNET cohort. **(A)** The number of patients (n = 13 total) with a site of extraesophageal EGID are represented using the immunodeficiency category assigned within the USIDNET at the time of data collection. **(B)** Representation of the location of gastrointestinal eosinophilia on a per-patient basis. CVID, Common variable immunodeficiency; CGD, chronic granulomatous disease; HIES, Hyper-IgE syndrome; SCID, severe combined immunodeficiency; IPEX, Immune dysregulation, polyendocrinopathy, enteropathy, X-linked, syndrome; EoE, eosinophilic esophagitis; EoG, eosinophilic gastritis; EoN, eosinophilic enteritis; EoC, eosinophilic colitis.

There were 13/74 (17.5%) patients in this cohort with eosinophilic gastritis (EoG), enteritis (EoN), and colitis (EoC). Most of these patients had CVID (38.4%) or combined immunodeficiencies (46%) (**Figure 2A**). Two patients (15.3%) had CGD, and a single patient had FOXP3-deficient immune dysregulation, polyendocrinopathy, enteropathy X-linked (IPEX) syndrome. Of the 13 patients with EGID, 3 had concomitant esophageal involvement while the other 10 did not (**Table 2**). Small bowel was the most reported site of eosinophilic inflammation in 84.6% (11/13) of the patients. Gastritis was the next most frequent, in 30.7% of patients. One patient with CGD (CYBB gp91-phox mutation) had involvement in the stomach, small bowel, and colon.

**Table 2:** Proportion of EoE and EGID in USIDNET patients.

	Number of patients in USIDNET	EoE patients, *n* (%)	EGID patients, *n* (%)
Total USIDNET database	5,484	61 (1.1%)	13 (0.2%)
Common variable immunodeficiency	1,820	27 (1.5%)	2 (0.1%)
Chronic Granulomatous Disease	570	4 (0.7%)	2 (0.4%)
Combined immunodeficiency	106	5 (4.7%)	2 (1.9%)
Hyper-IgE Syndrome	104	4 (3.8%)	2 (1.9%)
Autoimmune lymphoproliferative disorder	34	5 (14.7%)	none

When compared to the 5,484 overall USIDNET database at the time of dataset collection, EoE was present in 1.1% of patients and non-EoE EGID was present in 0.2% of patients (**Figure 2**). The database had 56% male participants, essential identical to the proportion of males in our cohort (56.7%, **Table 1**).

**Figure 3:**
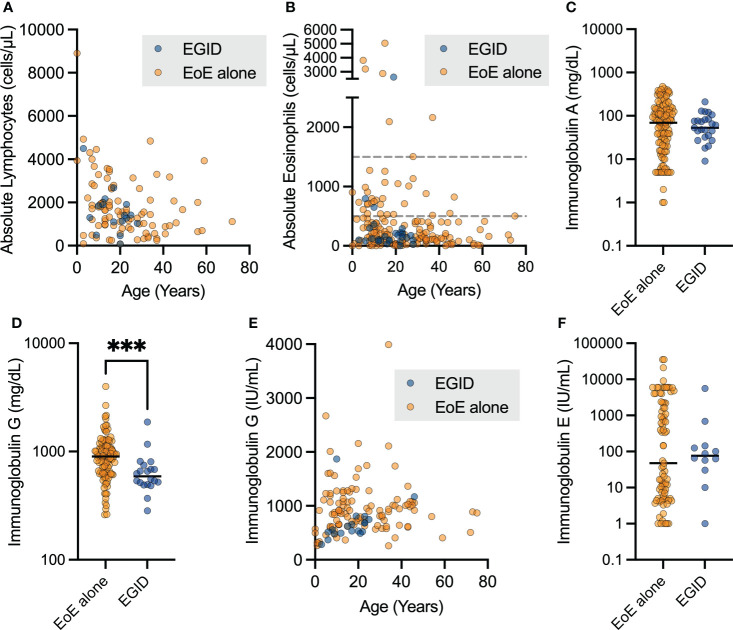
Patients with non-EoE EGID have lower uncorrected immunoglobulin G levels. The aggregate lab values for patients with EoE and non-EoE EGID are shown for the **(A)** absolute lymphocyte count and **(B)** absolute eosinophil count from CBC with differential measured in cells per microliter (dashed lines indicate 500 and 1500 cells per μL). **(C, D)** Serum immunoglobulin G levels in mg/dL were significantly lower in patients with EGID compared to those with EGID alone (***p=0.0008, Mann-Whitney U test) and **(E)** showed expected age-related increase over childhood years. Patients on immunoglobulin in replacement therapy were excluded from this analysis. **(F)** Serum immunoglobulin E levels were not significantly different between patients with EGID and EoE, and a subset of patients had significantly elevated IgE.

We examined the laboratory outcomes in relation to the diagnosis of EoE and EGID in this cohort. We did not see differences in the absolute lymphocyte count (ALC, **Figure 3A**) or absolute eosinophil count (AEC, **Figure 3B**) based on if the patients had EGID or only EoE. Nineteen patients (25%) had some degree of documented eosinophilia, with AEC ≥ 500 cells per microliter. Severe eosinophilia was not a feature in this cohort, and only one patient had AEC ≥ 5,000 cells per microliter.

We sought to examine antibody production due to its relevance to humoral immune protection and the pathogenesis of atopic disorders. Immunoglobulin A (IgA) production was not significantly different between patients with EoE and EGID (**Figure 3C**), however some patients did have low IgA as expected with diagnosis of IEI. We assessed the immunoglobulin G (IgG) levels of patients with EoE compared to those with distal GI EGID, excluding all laboratory values of patients noted to be on immunoglobulin replacement to avoid potential confounding due to therapy (**Figure 3D**). We observed a significantly lower serum IgG level in the EGID patients compared to those with EoE alone. This was potentially attributable to the GI loss of immunoglobulin. However, we are unable to correlate the lower immunoglobulin levels with periods of active EGID using the current dataset because USIDNET does not contain information about EGID disease activity. As expected, we observed that there was an age-related increase in serum IgG during childhood (**Figure 3E**). However, due to the small number of patients within the cohort, we did not perform additional analyses by age.

We next examined the serum IgE level within the cohort. There was no significant difference between patients with EoE and EGID (**Figure 3F**). Among the EoE patients, we observed distinct groups with both low- and high-serum IgE groups within the cohort. There were no significant differences in serum IgE based on age. Additional data regarding some labs of interest in EGID, including serum IgG4, and allergen-specific IgE were not available on patients within this cohort. Enumeration of CD19+CD27+IgM- switched memory B cell populations is available for some USIDNET patients. However, only one patient within this cohort had this data recorded.

**Figure 4:**
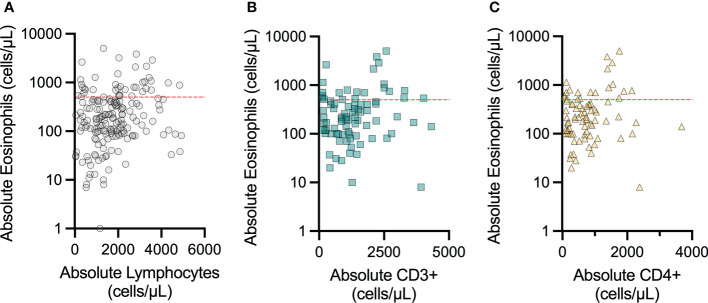
Peripheral eosinophil count does not vary with peripheral lymphocyte count in patients with concomitant IEI and eosinophilic GI disorders. The correlations between peripheral blood absolute eosinophil count and **(A)** peripheral blood absolute lymphocyte count, **(B)** peripheral blood absolute CD3+ count, and **(C)** peripheral blood absolute CD4+ count were not significant. The red dashed line indicates 500 eosinophils per microliter, which is clinically considered the initial cutoff for eosinophilia.

The association between lymphopenia and restricted T-cell repertoire and severe eosinophilic disease is well-established, and this inflammation can involve the GI tract (18, 19). Therefore, we examined lab values obtained for each patient at a single assessment within this cohort to determine if peripheral eosinophil count was correlated with T-cell counts. We correlated eosinophil count to lymphocyte count as well as absolute CD3+ and CD4+ counts, when available (**Figure 4**). In each instance, the AEC appeared unrelated to the peripheral ALC, and absolute CD3+ and CD4+ counts. GI biopsy eosinophil count data were not available for this patient cohort.

**Figure 5:**
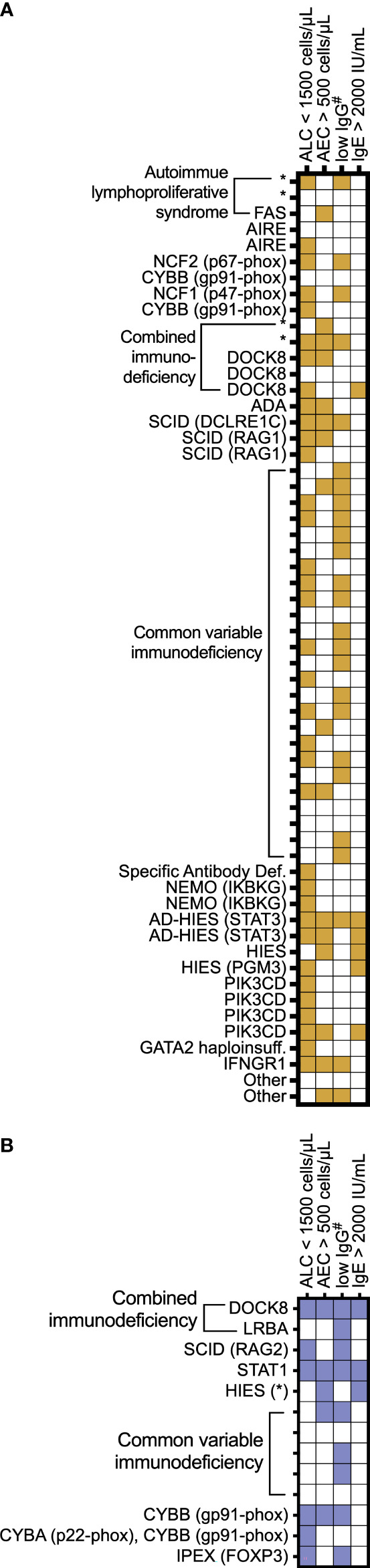
Individual patient IEI presentation. A subset of IEI patients with **(A)** EoE and **(B)** EGID are represented by IEI category as assigned in the USIDNET and by genetic diagnosis when known. The features are shaded for each patient if the lab values were recorded documenting the following: absolute lymphocyte count (ALC) < 1,500 cells/μL; absolute eosinophil count (AEC) > 500 cells/μL; #serum immunoglobulin levels < 700 mg/dL in patients 5 years of age and older or <500 in patients 4 years of age and younger; and serum immunoglobulin E (IgE) > 2,000 IU/mL. * denotes genetic diagnosis unknown.

We examined manifestations of IEI among the individuals in this cohort (**Figure 5**). We focused on common laboratory findings that may represent early findings suspicious for immunodeficiency, including serum IgE > 2000 IU/mL, AEC > 500 cells per microliter, and ALC < 1500 cells per microliter. Due to age-related differences in serum IgG levels, we noted patients with low serum IgG if it was less than 500 mg/dL in patients four years of age or under at the time of lab draw or less than 700 mg/dL in patients in patients five years of age and older at the time of lab draw. Of the 61 patients with EoE, 54 patients (88%) had recorded laboratory findings meeting these criteria (**Figure 5A**). In the EGID cohort, 11 of 13 patients had recorded lab abnormalities meeting these criteria. Due to ascertainment bias, it is possible that other patients in the cohort could have these lab findings, but they were not included in reports to USIDNET. Among this small cohort, we observed that the majority of patients presented with previously described immunologic findings consistent with their IEI and that there are multiple manifestations of IEI between different patients with similar diagnoses.

Next, we examined data regarding infectious complications across the cohort. These complications did not differ from previously described infectious complications of patients with the underlying IEI, with the exception that roughly one-fourth of the cohort (17/74 = 22.9% patients) had experienced oral or esophageal thrush. Of these patients, three had SCID (1 Artemis, 2-RAG1), four had CVID, three had AIRE, one had DOCK8 deficiency, one had IPEX syndrome, one had CGD, one had STAT1, and one had PIK3CD. Esophageal thrush and candidiasis are known complications of swallowed topical steroid therapy in EoE and have been reported in up to 8.7% of patients on swallowed steroid therapy (20). Patients on swallowed budesonide therapy may have asymptomatic esophageal candidal infection noted at the time of an endoscopy planned for EoE follow-up. However, our cohort data did not specify if these candidal infections predated the diagnosis of EoE or occurred after the EoE diagnosis, and also, the data lacks the ability to ascertain if these episodes were related to swallowed steroid therapy for EoE. Five patients had complications of esophageal stenosis/stricture noted in their USIDNET record.

GI tissue eosinophilia is associated with several disease states that can complicate hematopoietic stem cell transplantation, including infections, graft versus host disease (GVHD), and diagnosis of primary EGID (21–26). Differentiating these clinical entities is essential both to determine effective treatment and because the diagnosis of primary EGID requires the exclusion of other probable causes of GI tissue eosinophilia (27, 28). Esophagitis is a reported complication of bone marrow transplantation and can be infectious or secondary to GVHD. A new diagnosis of EGID, including EoE, has been described in patients following hematopoietic stem cell transplantation (24–26). Our cohort included 15 patients that had undergone a stem cell transplant for the management of IEI, and 11/71 (15.4%) of the patients’ transplant statuses were unrecorded. No patients in the cohort were noted to have a complication of GVHD, and the timing of the transplant versus EGID disease onset was not available.

## Discussion

There has been a growing awareness that IEI presents with immune dysregulation, and specific IEI carry an increased risk for atopic disorders (5–7). However, there have been few reports about the association of IEI with EGID, and it is not well-understood which IEI may present with EoE as a feature. In this study, we identified a cohort of 74 IEI patients in the USIDNET, with a diagnosis of EoE alone or EGID of one or more distal sites. Although there have been case or small cohort reports regarding EGIDs in IEI patients, our study utilizes the USIDNET to broadly ask which IEI patients have been diagnosed with EGIDs. In identifying 61 EoE patients and 13 EGID patients, our results (**Table 2**) suggest that EGIDs within the context of IEI may be more common than initially suspected. The population prevalence of EoE is currently estimated at 0.5-1 in 1000 whereas non-EoE EGIDs have been reported at 3-8 per 100,000 (10, 29). From the 5,484 total patients in the USIDNET at the time of our study, we might therefore have expected a range of 5 to 11 EoE patients and perhaps 1 patient with non-EoE EGID. A limitation of this study is that we cannot account for ascertainment and other biases that may enrich the diagnosis of EGIDs in this cohort and it would be beneficial for additional studies to be performed to validate this finding in independent cohorts. However, it is plausible that EGIDs could be enriched in IEI patients given that multiple IEI have been associated with immune dysregulation, including atopic manifestations and inflammatory bowel disease.

Patients in this study cohort were diagnosed with diverse underlying IEI, including CVID and multiple specific monogenic gene defects causing IEI. While there have been a handful of reports detail EGID in IEI patients (11–13, 15, 16), our study highlights that EoE and non-EoE EGID occur in the context of several categories of IEI that had previously been unrecognized (ie: interferonopathies and ALPS). There have been prior case reports of EoE in CVID patients (11, 12), but we identified 24 CVID patients with EoE and 2 with EGID, suggesting that EGID within the context of CVID may be more common than previously suspected.

Although a large proportion of our patient cohort had CVID, we did not identify EGID or EoE patients in the database with other intrinsic B cell defects like X-linked agammaglobulinemia or Hyper-IgM syndrome. Other diagnoses including complement deficiencies and innate immune defects were also not represented. One limitation to our methods is possible ascertainment bias in recording patient data in the USIDNET, and that both IEI and EGID are uncommon diagnoses. Therefore, it is necessary to interpret this finding with caution.

Eosinophilic infiltration of the esophagus has been reported in 11 STAT3 HIES patients (15). Consistent with prior reports, we identified 5 patients in this cohort with HIES, including 2 patients with autosomal-dominant STAT3-loss of function, one with PGM3 loss of function, and two with undefined gene defects. As in prior studies, IgE levels were not uniformly elevated across these patients, ranging from 343 IU/mL to 35,451 IU/mL, with a median of 5,918 IU/mL (**Figure 3E**). This finding emphasizes the need to screen for other clinical features in addition to laboratory values. There are case reports of therapeutic success using the anti-IL4Rα monoclonal antibody dupilumab to control eosinophilic GI inflammation in HIES patients (30, 31).

The pathogenesis of EoE and EGID is distinct and complex, involving multiple inflammatory effector mechanisms (32, 33). EoE, EoG, EoN, and EoC each have recommended diagnostic criteria based on the number of eosinophils present on histopathology from the GI biopsy tissue (34). The information available in USIDNET is based on clinical diagnoses and we were unable to review patient records or pathology reports or images for this study. Specific information about EGID diagnoses including the numbers of biopsies and biopsy criteria used for diagnosis, and presences of confounding conditions including infections, GVHD, or other GI conditions were not able to be evaluated. This is an important limitation and raises several questions for future studies. One criterion for diagnosis of EGID is exclusion of secondary causes of gastrointestinal eosinophilia (27, 28), however the patients in this cohort have unique risks for GI conditions associated with tissue eosinophilia including candidal infections, GVHD, and inflammatory bowel disease (IBD). IBD can initially present with eosinophilic infiltrates (35, 36), and this is frequently seen in patients with CGD (37). Among our cohort, 5 patients had concurrent EoE and IBD, including one patient with CGD SCID, CVID, and IPEX and one within the “other” category. EoE is increasingly recognized to co-occur with inflammatory bowel disease (38–40), and in some cohorts, the patients are more likely to be male, present at a younger age, and be associated with comorbid atopic disorders (41). It is important to note that mucocutaneous candidiasis can cause secondary gastrointestinal eosinophilia (13, 27, 28). Patients with some forms of IEI, like HIES or combined immunodeficiency, are more susceptible to candidal infections. Differentiating these clinical entities is critical to establish accurate diagnosis of EGID. It would be beneficial if future studies of EGID in IEI were conducted in contexts where trained pathologists could review biopsy slides to verify the accuracy of EGID diagnoses and perform standardized evaluation to determine if histologic differences are evident in IEI patients.

There were 7 patients with combined immunodeficiencies and 5 patients with SCID across this cohort. Lymphopenia has been linked with peripheral and tissue eosinophilia (18, 19), which has been linked to restriction in the repertoire capacity of conventional and regulatory T cells and a shift to T2-phenotype. We did not see a correlation between overall lymphocyte or peripheral CD3+ or CD4+ and eosinophil counts in this cohort (**Figure 4**). USIDNET does not have patients’ EGID disease status (ie: active vs. quiescent) at the time of laboratory testing, which may impact peripheral eosinophil values for EGID patients. The dataset also lacked information regarding T-cell receptor diversity within the CD4+ subset. Therefore, we cannot assess if restricted T-cell receptor repertoire diversity correlated with eosinophilia in these subjects.

EGIDs, including EoE are generally thought to be complex disorders arising from environmental exposures in susceptible individuals. However, in rare cases monogenic susceptibility has been demonstrated (42–46), including links to syndromic disorders including PTEN Hamartoma syndrome, connective tissue disorders, and Netherton syndrome. Our data suggests that EGID may be more common in patients with IEI than would be expected based on estimates of prevalence in the general population. More work is needed to validate these findings in additional patient cohorts, and to understand the extent to which co-morbid diagnoses of IEI and EGID impacts EGID outcome and therapeutic response. Patients with EGID and IEI may have distinct clinical features, comprising a unique endotype of disease.

## Data availability statement

Requests to access the datasets should be directed to USIDNET.org. The USIDNET.org primary immunodeficiency patient registry database contains validated, de-identified patient data which can be released for research purposes.

## Ethics statement

The studies involving human participants were reviewed and approved by USIDNET is an Immune Deficiency Foundation program and is an NIH-funded research consortium that maintains a patient-consented registry of data from PID patients in North America. Patient data collection for USIDNET proceeds under the supervision of each enrolling institution’s Institutional Review Board. All patients or their legal representatives provide written informed consent for inclusion into the USIDNET database, which contains clinical, laboratory, and molecular data from PID patients. The patients (or parents) consented directly.

## Author contributions

LG, JS, and MR conceptualized study; EG, RF, KS, and JP recruited patients and USIDNET study execution, MR, PT, JS, and KS data analysis; PT and MR, original draft. All authors participated in review and editing of the manuscript and agree to be accountable for its contents.

## Funding

The U.S. Immunodeficiency Network (USIDNET), a program of the Immune Deficiency Foundation (IDF), is supported by a cooperative agreement, U24AI86837, from the National Institute of Allergy and Infectious Diseases (NIAID). MR is funded by NIH K08AI148456, and KS is funded by The Wallace Chair of Pediatrics. The content is solely the responsibility of the authors and does not necessarily represent the official views of the National Institutes of Health.

## Acknowledgments

We gratefully acknowledge additional USIDNET Consortium enrolling physicians, including Niraj Patel MD, Rebecca Buckley MD, Vivian Cino CRNP, Charolotte-Cunningham Rundles MD, PhD, Daniel Suez MD, Elie Haddad MD, PhD, Elizabeth Secord MD, Heather Lehman MD, John Routes MD, and Ralph Shapiro, MD.

## Conflict of interest

JP receives royalties from UpToDate and her spouse is employed by Invitae, a DNA sequencing company.

The remaining authors declare that the research was conducted in the absence of any commercial or financial relationships that could be construed as a potential conflict of interest.

## Publisher’s note

All claims expressed in this article are solely those of the authors and do not necessarily represent those of their affiliated organizations, or those of the publisher, the editors and the reviewers. Any product that may be evaluated in this article, or claim that may be made by its manufacturer, is not guaranteed or endorsed by the publisher.
